# The causal nexus between imports and economic growth in China, India and G7 countries: granger causality analysis in the frequency domain

**DOI:** 10.1016/j.heliyon.2022.e10180

**Published:** 2022-08-10

**Authors:** Khalid Usman, Usman Bashir

**Affiliations:** a School of Economics and Management, Guangzhou City Construction College, Guangzhou City, Guangdong Province, PR China; b Department of Economics and Finance, College of Business Administration, University of Bahrain, P.O Box 32038 Sakhir, Kingdom of Bahrain

**Keywords:** Imports and economic growth, Time domain, Frequency domain, BC granger Causality, China, India and G7 countries

## Abstract

This research examine the causal nexus between imports and economic growth in China, India and G7 economies. We use BC granger causality analysis in the frequency domain short (temporary) and long term (permanent) causality. Our results suggested that, there is a frequency domain (high and low) proof of the bi-directional causal nexus between imports and economic growth. The imports and economic growth in most economies seem to be long-term and short-term dependent. This empirical study shows that policymakers of these big economies need to analyze the transformation in the import-economic growth causality robustness throughout the year when planning policy actions.

## 1 Introduction

A country frequently requires to import with the purpose of obtain transitional productivity inputs and manufactured merchandise for national productivity and national consumption, respectively. “Imports led growth (ILG)” shows that economic growth might be mainly determined by imports growth. “Endogenous growth model” indicates that imports can be the medium for long-term economic growth, as it gives local enterprises the opportunity to obtain the required intermediary and international high-tech (Coe and Helpman, 1995). Import growth may act like a channel in order to shift of growth promoting global R&D expertise from rich to poor economies (Lawrence and Weinstein, 1999; Mazumdar, 2000). As a medium to transmit technology and innovation, imports provide accessibility to wealth and intermediate products, and enhance challenges to make sure a persuasive allocation of resources (Tsegaye, 2015).

Imports initiate spillage in the circular stream of revenue, which is considered to be the main reason of unemployment rather than economic growth (Liu et al., 1997). As a result, certain countries shield domestic markets from rivalry with international manufacturers. However, the endogenous growth model shows that imports are crucial in a country's growth process. When imports collated with exports, it revealed the leading role of imports in economic growth, thus growth is export-oriented and imports are export-oriented (Awokuse, 2007, 2008); (Shan and Sun, 1998bib_Awokuse_2008). The trend of import driven economic growth has further emerged as domestic import substitution businesses struggle with international rivals in developed countries (Kim et al., 2007).

To making good trade polices it’s very important to understand the causal nexus between imports and economic growth. For example, if imports trade fails to support economic growth, countries may be more willing to follow import substitute trade policy. However, the implementation of import substitute policy may promote economic growth. After eliminating import substitute policy and utilizing import liberalization trade policy, the largest part of Asian economies cautiously feel the favorable cause of import (Nguyen, 2011). According to Kim et al. (2007), they believe that imports liberalization policies promote the growth of an economy.

Previous studies used variations of the Granger causality (GC) across the “time-domain” to present facts of causal correlation between imports and economic growth, which purport not so exhortative because they couldn’t explain the possible modifications in the dynamics of causality in the long term. The GC test in time domain did not consider the possible change of causality dynamics with time, because they strictly assumed the linearity of vector auto-regression (VAR) system Opoku et al. (2019).

The “frequency domain or spectral Breitung and Candelon (BC) causality test”, created by Breitung and Candelon (2006), related to the prior study of Hosoya (1991) and Geweke (1982). The major dissimilarity between the time domain and the Spectral or frequency domain approach is that the "time domain" demonstrates when a definite change happens between the time-series, while the "frequency domain" evaluates the level of a definite change in the time-series. In a short-series of analysis, serial patterns perhaps vital and the frequency domain allows the exclusion of these variations. In addition, the frequency-domain approach allows examining nonlinear and causal cycles, i.e., causal relationships at high or low frequencies (Gokmenoglu et al., 2019). However, some scholars investigate the causal effects between energy and economic, according to Sivaram and Saha (2018), energy is the means of support of countries economy, and a permanent energy supply is important for feasible economic growth and state security. Su et al., 2021a, Su et al., 2021b studied the linkage between geopolitical risk and renewable energy. Their results demonstrated that there was no causal nexus between geopolitical risks and renewable energy. On the other hand, the results crosswise different sub-samples illustrated the bidirectional causal relationship between geopolitical risk and renewable energy. Geopolitical risks have both positive and negative impacts on renewable energy, indicates that geopolitical risk plays a vital part in the development of renewable energy. In addition, the global economic growths, technological process in fossil fuels and the continuous improvement of environmental engagements have fostered the growth of renewable energy, which accommodates novel forces and hikes rivalry.

Therefore, advanced fiscal organisations are a way to promote growth in economic and get rid of the curse of rent resource (Shahbaz et al., 2018a,b). Zaidi et al. (2019), used the panel data on OECD economies from 1990 to 2016 to analyze the impact of human capital (HC), natural resources, globalization and economic growth on financial development and predicted that economic growth, capital formation; globalization, natural resources and human capital had a significant impact on financial development. Human capital imparts to the productive resort natural resources, the consistency of the financial system and economic growth (Becker, 2009; Zaidi et al., 2019). J. Guan et al. (2020), further thoroughly investigated the linkage between the financial development and natural resources rents, systematically and analytically, so as to supply to the expansion of financial growth policies in China. Their research explored the cause of natural resources rents, economic growth, human capital, and globalization on financial development from 1971 to 2017. The findings of their empirical research presented notable proof for the nexus between financial development and its factors are co-integrated. Their results proposed that there is a resource curse in China, because research has proved that natural resources rents are the main indicators for the corrosion of financial development, because they will contribute to short and long-run financial growth.

With the implementation of maritime power strategy, numerous kinds of studies investigate the linkage between financial development and marine economic growth of China (He et al., 2019; Tian et al., 2019). However, Su et al., 2021a, Su et al., 2021b investigated the causal relationships between China’ financial development and marine economic growth. The influences of financial development on marine economic growth differ from province to province. Furthermore they studied the influence of regional dissimilarities on the comprehensive marine economic growth; the results indicated that there were substantial regional dissimilarities in the causality of the three marine industries. Zhang et al. (2021) explore the causal relationship between carbon dioxide (CO_2_) emission and GDP to investigate the efficacy of the EKC theory in P.R. China. The study utilized full sample granger causality test, The findings demonstrated that the GDP had positive impact on carbon dioxide (CO_2_) emission, but the carbon dioxide (CO_2_) emission have no affect on GDP. On the other hand, there were not causal effects between carbon dioxide (CO_2_) emission and GDP growth. Research scholars and experts believe that the key issue of greenhouse gas emissions, especially CO_2_ emissions is global warming. Umar et al., 2020a, Umar et al., 2020b and Zhang et al. (2021), concluded reverse U-shape linkage between the GDP per capita, and the emission per capita of three contaminant. As a result, a long term causal linkage between GDP and use of energy, while this was not the case in China. Yuan et al. (2008) and Umar et al., 2020a, Umar et al., 2020b, As the most populous country on the earth, the results showed that China is one of the countries with the biggest contributor of carbon dioxide (CO_2_) in the environment (Umar et al., 2020a, Umar et al., 2020b). A general opinion is that the economic growth and energy consumption are majority significant factors that influencing carbon dioxide (CO_2_) emissions (Zhu et al., 2016; Cevik et al., 2020; Shakeel and Ahmed, 2021; Umar et al., 2021).. Therefore, the other study direction involved the causality test between carbon dioxide (CO_2_) emissions and economic growth. The scientific research results showed ambiguity, because there is no integrated finding on the linkage between carbon dioxide (CO_2_) and economic growth (Umar et al. (2020).

Aluko and Obalade (2020), studied the relationship between imports (imports of goods and services (US$)) and economic growth (GDP) in a sample of 26 African economies from 1990 to 2015 under the umbrella of neoclassical production function. Using Toda &Yamamoto (1995) Granger non causality test, their research findings illustrate that there is no causal effects between import and economic growth in more than half of the countries, which proves that the causality is not exist between import and economic growth. Correspondingly, Aluko and Adeyeye (2020) tested the causal relationship between imports and economic growth in 41 African economies. Their research findings show that the uni-directional causality exists between import and economic growth, and the uni-directional causality found between economic growth and import in a few countries, the neutrality hypothesis is effective in the short and long term in most countries. Likewise, Maitra (2020) examined India's ILG hypothesis in the post reform era. His study discovered significant proof confirming the ILG hypothesis in the short and long term, indicating that imports have an important influence on India's economic growth.

We use time series model to study the causal nexus between imports (imports of goods and services (current US dollar), and economic growth (gdp per capita (constant 2010 US dollar) of China, India and G7 countries, so as to increase the insufficient empirical facts of the countries. We test the Granger causality between imports and economic growth using the frequency domain method, which presents new proof for the scientific literature. In the frequency domain, we use Breitung and Candelon’s (2006), BC Granger causality test, which enables us to identify the causal effect between imports and economic growth in different period in the framework of bivariate VAR model. It is essential to execute GC test in the frequency domain Lemmens et al. (2008), consequently, it also be able to produce new additional understanding into causality as compared with the conventional Granger causality test.

Because of trade cycles, policy and radical changes, robustness of causal correlations between imports and economic growth may change after some period of time. Therefore, through the use of spectral analysis or frequency domain approach of GC, it’s extremely vital to elucidate the potential changes of the causality robustness between imports and economic growth ultimately. Our research is mainly helpful for policymakers to applicable the short-term and long-term causal linkage between import and economic growth in China, India and G7 (Canada, France, Germany, Italy, Japan, UK, USA) economies under analysis.

The rest of this article is as follows. Section 2 consists of literature review. The methodology and data collection are discuss in Section 3. Section 4 and 5 discusses the results of the analysis, and presented conclusion.

## 2 Literature review

The plethora studies present facts to observe the role of imports and economic growth, and export analysis -export growth hypothesis. Awokuse (2007), referred that neglecting the role of imports and exaggerating on exports as the major factor on growth may mislead or be insufficient for growth analysis. Yet, if there is a one-way correlation between exports and imports, it depends on import growth (). Hence, ignoring imports in the analysis may decline the factual trade influence on economic growth. High income countries demonstrate the imports-led growth hypothesis, while lower income economies demonstrate two-way correlation (Islam et al., 2012). If economies have satisfactory foreign exchange reserves, economic growth possibly is reinforced by importing goods and services for consumption and manufacture purposes (Baharumshah and Rashid, 1999).

The causality between the variables can be classified by four hypotheses: (1) imports-led growth (ILG) hypothesis (2) growth-Led imports (GLI) hypothesis (3) feedback (FB) hypothesis and (4) neutrality (NH) hypothesis. According to the ILG hypothesis, any economy imports fix assets and high-tech products, which added results in the expansion of industrial infrastructure and alternately stimulate economic growth (Hanson and Trewavas, 1982). The ILG hypothesis medium ascertains long-term economic growth, thus it presents the essential intermediate raw materials and universal cutting-edge technology for domestic industries (Coe and Helpman, 1995).

On the other hand GLIH hypothesis holds that economic growth boosts import desire, indicating that imports are triggered by economic growth (EC). Such proposition/hypothesis carries that the control of causal relationship following economic growth to import. The FB proposition/hypothesis shows that the GLI hypothesis and the ILG hypothesis prevail as well. This paper points out that two-dimensional causal correlation between import and economic growth exist, which shows that import and economic growth promote together. Nevertheless, the neutral hypothesis is just the converse of the feedback hypothesis, because it does not support the ILG hypothesis or the GLI hypothesis as well. However it shows that there will have no causation between import and economic growth variables. The facts supporting the above hypothesis have been demonstrated in experimental studies. Thereby peculiarity of some studies, a significant methodological technique pointed out in the research articles is the utilization of granger causality test.

Our research is especially valuable for decisions makers to understand the short (temporary) and long term (permanent) robustness of causal nexus between imports and economic growth in China, India and G7 (Canada, France, Germany, Italy, Japan, UK, USA) economies. We will utilize the time series modelling technique to analyses the causality effect between imports and economic growth of China, India and G7 member countries, therefore extending the sparse empirical facts of most of the countries under analysis. The “frequency domain” method practiced to inspect the GC causal linkage between imports and economic growth variables; we provide new facts for the literature.

We utilize the GC test of Breitung and Candelon (2006) in the “spectral or frequency domain” enables to identify the causal robustness between imports and economic growth variables in different time duration in the model of bi-dimensional VAR model. Lemmens et al. (2008), believe that it is remarkable to carryout GC test in frequency domain, thus it possibly generate novel supplementary intuitions on causality compared with traditional Granger causality test. The facts supporting the above hypothesis have been recorded in an empirical revision. In addition to several studies, the highlighting methodological approach examined in studies is to apply the Granger anchorage test. We show empirical studies in Table 1.

**Table 1:** List of empirical studies and supporting hypothesis.

Study	Time period	Countries	Methodology	Evidence/Hypothesis
Olufemi Adewale Aluko & Patrick Olufemi Adeyeye (2020)	1985–2017	41- countries from South African	Toda-Yamamoto (T-Y)GC test	ILG, GLI, FB,Neutrality
Fapetu, O.,& Owoeye.S.D. (2017)	1981–2014	Federal Republic of Nigeria	Toda-Yamamoto (T-Y)GC test	ILG
Bakari,S. (2017)	1965–2015	Arab Republic of Egypt	VECM GC test	ILG
Bakari,S. & Krit, M.(2017)	1960–2015	Mauritania	VECM GC test	FB
El Alaoui, A. (2015)	1980–2013	Kingdom of Morocco	VECM GC test	FB
Andrews, A.P. (2015)	1970–2011	Republic of Liberia	Pairwise GC test	FB
Kumari, D.,& Malhotra, N. (2014)	1980–2012	India and China	Toda-Yamamoto (T-Y)GC test	FB and Neutrality
Chang et al. (2014)	1996–2011	9- provinces of South African	Bootstrap panel Granger causality test	GLI, FB, Neutrality
Hye et al. (2013)	1960–2009	6-South Asian countries	Modified Granger causality test	FB
Rahman, M.M., and Shahbaz, M. (2013)	1990–2010	Pakistan	VECM GC test	FB
Hye, Q.M. (2012)	1978–2009	People’s Republic of China	ARDL modelling approach	FB

## 3 Methodology and data collection

Our study is on the bases of annual data for the time of 1978–2019, preferred on the basis of the availability of fully data covering economic growth and imports of China, India and G7 countries. The data is collected from the World Bank website and we use imports (imports of goods and services (current US dollar), and economic growth (gdp per capita (constant 2010 US dollar) accordingly. We convert the data through natural logarithm to obtain superior distribution characteristics (Ciner 2011).

## 4 Empirical test approaches

In frequency domain, we practice Breitung and Candelon (BC) Granger causality test, which show the causal prototype about two factors at distinct frequency domains by applying linear restrictions through auto-regression framework inthe two-dimensional vector auto-regression model (VAR) (Granger, 1969; Geweke, 1982; Hosoya, 1991; Breitung and Candelon 2006; Ciner, 2011). First, we suppose *Y*_*t*_*= (EG*_*t*_*, IMP*_*t*_*)*’ be a bi-directional matrix of time series length *t = 1, …., T.* In the following section, *EG*_*t*_ and *IMP*_*t*_ interpretation for economic growth and imports correspondingly. We supposed that *Y*_*t*_ has a finite order (Eq.1) vector auto-regression (VAR) process Breitung and Candelon (2006).

(1) $$\Theta {\left(L\right)}_{Y_{t}}=\varepsilon_{t}$$

Where Θ(L) = I – Θ_1_L – ······ – Θ_p_L^p^ is a 2 × 2 lag polynomial with the back-shift operator *L*^*k*^*Y*_*t*_*= Y*_*t-k*_*.* The vector error ${\text{ε}}_{\text{t}},$ is white-noise with 0 mean, and *E(*$\varepsilon_{t}\varepsilon \prime_{t})$ *=Ʃ*, where Ʃ is positive definite covariance matrix.

Implementing Cholesky decomposition, *G’G= Ʃ-1* (where G be the lower three dimensional matrix) such that E (η_t_ή_t_) = I and η_t_ = G ${\text{ε}}_{\text{t}}$. In case of stationary system, the MA, demonstration of the system is as Eq. (2):-

(2) $$Y_{t}=ф\left(L\right)\varepsilon_{t}=\left[\begin{array}{ll} {ф}_{11}\left(L\right) & {ф}_{12}\left(L\right)\\ {ф}_{21}\left(L\right) & {ф}_{22}\left(L\right) \end{array}\right]\left[\begin{array}{l} \varepsilon_{1t}\\ \varepsilon_{2t} \end{array}\right]=\psi \left(L\right)\eta_{t}=\left[\begin{array}{ll} \psi_{11}\left(L\right) & \psi_{12}\left(L\right)\\ \psi_{21}\left(L\right) & \psi_{22}\left(L\right) \end{array}\right]\left[\begin{array}{l} \eta_{1t}\\ \eta_{2t} \end{array}\right]$$

Where $\text{ф}\left(\text{L}\right)=\text{Θ}{\left(\text{L}\right)}^{-1}$ and$\text{ψ}\left(\text{L}\right)=\text{ф}\left(\text{L}\right){\text{G}}^{-1}$. Applying this illustration, the spectral density of $EG_{t}$ as Eq. (3):-

(3) $$f_{EG}\left(\varpi \right)=\frac{1}{2\pi }\left\{{\left|\psi_{11}(e^{-i\varpi })\right|}^{2}+{\left|\psi_{12}(e^{-i\varpi })\right|}^{2}\right\}$$

The evaluation of CM recommended by Geweke (1982) and Hosoya (1991) is classified as (Eqs. (4) and (5)):-

(4) $$CM_{EG\to IMP}\left(\tilde{\omega }\right)=\text{log ​}\left[\frac{2\text{π}{\text{f}}_{\text{x ​}}\left(\tilde{\omega }\right)}{{\left|{\text{ψ}}_{11}({\text{e}}^{-\text{i}\tilde{\omega }})\right|}^{2}}\right]$$

(5) $$=log\;\left[1+\frac{{\left|\psi_{12\;}(e^{-i\tilde{\omega }})\right|}^{2}}{{\left|\psi_{11\;}(e^{-i\tilde{\omega }})\right|}^{2}}\right]$$

The computation is zero if ${\left|{\text{ψ}}_{12}({\text{e}}^{-\text{i}\tilde{\omega }})\right|}^{2}=0$, wherein situation we state that *EG*_*t*_ fails to cause *IMP*_*t*_ at frequency $\tilde{\omega }$. In this frequency domain the calculation of causal relationship is possibly classified by applying the orthogonalized MA depiction Eq. (6):-.

(6) $$\tilde{\omega }Y_{t\;}=\tilde{ф}\left(L\right)\varepsilon_{t}=\tilde{\psi }\left(L\right)\eta_{t}$$

where $\tilde{\text{ψ}}\left(\text{L}\right)=\tilde{ф}\left(\text{L}\right){\text{G}}^{-1}$, ${\text{η}}_{\text{t}}$ = ${\text{G}}_{\text{εt}}$, and (where G be the lower three dimensional matrix), such that E (η_t_ή_t_) = I. Point out that in a bi-dimensional co-integrated system, $\overset{\prime }{\beta }\tilde{\text{ψ}}\left(1\right)=0$ where β is a co-integration matrix so that $\overset{\prime }{\beta },\;\tilde{{\text{y}}_{\text{t}}}$ is stationary. In this sense the stationary cause the following CM is Eq. (7):-

(7) $$CM_{EG\to IMP}\left(\tilde{\omega }\right)=log\;\left[1+\frac{{\left|{\tilde{\psi }}_{12\;}(e^{-i\tilde{\omega }})\right|}^{2}}{{\left|{\tilde{\psi }}_{11\;}(e^{-i\tilde{\omega }})\right|}^{2}}\right]$$

However, according to Yao and Hosoya (2000), in this hypothesis, *EG*_*t*_ fails to cause *IMP*_*t*_

at frequency $\tilde{\omega }$ we contemplate the null hypothesis among a bi-dimensional framework. Hosoya (2001), propose to asses ${\text{CM}}_{\text{EG}\to \text{IMP}}\left(\tilde{\omega }\right)$ (Eq. 8) by alternating $\left|{\text{ψ}}_{11}({\text{e}}^{-\text{i}\tilde{\omega }})\right|$ and $\left|{\text{ψ}}_{12}({\text{e}}^{-\text{i}\tilde{\omega }})\right|$ in Eq. (5) with assessments achieved from the fitted VAR.

(8) $$CM_{EG\to IMP}\left(\tilde{\omega }\right)=0\;$$

To check the null hypothesis, a basic technique suggested by Breitung and Candelon (2006). *EG*_*t*_ fails to cause *IMP*_*t*_ at frequency ῶ (Eq.9):

(9) $$\left|\Theta_{12\;}(e^{-i\tilde{\omega }})\right|=\left|\overset{p}{\underset{k=1}{\sum }}\theta_{12,k\;}\;\cos\left(k\tilde{\omega }\right)-\overset{p}{\underset{k=1}{\sum }}\theta_{12,k\;}\;\sin\left(k\tilde{\omega }\right)i\right|=0$$

Where ${\text{θ}}_{12,\text{k}}$ is the (1,2)-element of ${\text{Θ}}_{\text{k}}$. Therefore, essential and satisfactory conditions (Eq.10) for $\left|{\text{Θ}}_{12}({\text{e}}^{-\text{i}\tilde{\omega }})\right|=0$. As (Eq. 11), $\sin\left(\text{k}\tilde{\omega }\right)=0$ for $\tilde{\omega }=0$ and $\tilde{\omega }=\text{π}$, restriction can be dropped in these situations.

(10) $$\overset{\text{p}}{\underset{\text{k}=1}{\sum }}{\text{θ}}_{12,\text{k}}\;\cos\left(\text{k}\tilde{\omega }\right)=0$$

(11) $$\overset{\text{p}}{\underset{\text{k}=1}{\sum }}{\text{θ}}_{12,\text{k}}\;\sin\left(\text{k}\tilde{\omega }\right)=0$$

Consequently, the “null hypothesis” of no GC at frequency ῶ can be examined by applying a standard F-test for the linear restrictions. Which results *F(2,T-2p)* distribution, for every ῶ between 0 and π, with T being the several measurements in the series and VAR optimal lag order, correspondingly.

According to Lemmens et al. (2008), we use BIC criterion to apply the VAR optimal lag order for BC test in frequency domain. “According to Ciner (2011), we calculated the causal test statistics at higher frequency is (ῶ = 2.5), and lower frequency is (ῶ = .01). The GC test of higher frequency and lower frequency is correspondence to short-term and long-term BC test”.

As discussed previously, there were four hypotheses which could be tested in the imports and economic growth (short and long term) of China, India and G7 countries. These four hypotheses are: (1). Imports-led growth (ILG) hypothesis (2). Growth-Led Imports (GLI) hypothesis (3). Feedback (FB) hypothesis (4). Neutrality (NH) hypothesis.

For comparability, we apply time domain GC test under Toda and Yamamoto (1995). GC test fails to rely on the pretesting for co-integration and unit root test when testing the causality of power and sample size. GC test shows that “optimal lag order” of VAR model optimizes Akaike information criterion.

## 5 Empirical results

The initial stage of our study to covers the test of the integration order of variables. In this regards, we apply Zivot and Andrews (Zandrew) unit root test, which implies a structural break in trend/intercept or both (Zivot and Andrews, 1992). The optimal lag order and the length of integration of variables carrying out the unit root test are subjected to means of Bayesian information criterion (BIC) presented in appendix 1. We consider that, the order of integration for every country are mixed, either I (0) and I (1), or just I (0) or I (1).

It’s difficult to conclude the integration order in different countries. For this reason, we adhere a additional variables technique, recommended by Toda and Yamamoto (1995) and Dolado and Lütkepohl (1996), thus we augment with single or different additional lags order of the VAR model, so as to control the influence that perhaps resulting from non-stationary variables in the VAR model.

The second stage of our empirical study comprises in testing the granger causality between imports and economic growth for China, Indian and G7 countries. Effectively carrying out our hypothesis, we show the granger causality results in the time domain before proceeding the assessment in the frequency domain granger causality.

Table 2 exhibits the effects of GC tests between imports and economic growth for China, Indian and G7 countries are summed up as follows. Our findings in the time domain causal test elaborate that bi-directional causality exist between imports and economic growth for seven economies (China, India, Canada, Germany, Japan, Italy and USA), hence the feedback hypothesis. However, we found uni-directional causality between economic growth to imports for two countries (France and UK), thus justifying the growth led import hypothesis. However, our empirical results failed to provide any evidence of neutrality hypothesis and ILG hypothesis in time domain granger causal test in China, India and G7 countries.

**Table 2:** Results of time domain Granger causality test.

Study	Time period	Time domain GC test				
		Imp → Real		Real → Imp		hypothesis
China	1978–2019	13.662	(0.001)∗	12.427	(0.002)∗	FBH
India	1978–2019	10.949	(0.012)∗∗	9.4436	(0.009)∗	FBH
Canada	1978–2019	6.7801	(0.034)∗∗	.28797	(0.866)∗∗∗	FBH
France	1978–2019	2.2703	(0.518)	12.477	(0.006)∗	GLIH
Germany	1978–2019	5.6619	(0.059)∗∗∗	12.427	(0.002)∗	FBH
Japan	1978–2019	7.7803	(0.020)∗∗	8.3189	(0.016)∗∗	FBH
Italy	1978–2019	.37306	(0.946)∗∗∗	28.327	(0.000)∗	FBH
UK	1978–2019	3.3523	(0.501)	11.887	(0.018)∗∗	GLIH
USA	1978–2019	5.9901	(0.200)∗∗	9.7586	(0.045)∗	FBH

Note: - Number in parentheses is p-values. ∗, ∗∗, ∗∗∗, denotes significant at 1%. 5% and 10% level respectively. Imp denotes (imports of goods and services (current US$), Real denotes (gdp per capita (constant 2010 US$).

Hereafter, checking the granger causal test between imports and economic growth in time domain, we now utilize the granger causal test in the frequency domain. Table 3 exhibits the outcomes of granger causal test in frequency domain between imports and economic growth in Indian, China, and G7 countries. However, frequency domain BC granger causality test states different outcomes between imports and economic growth. Firstly, we perceive that there are bidirectional causality (permanent and temporary causality) moving between imports and economic growth for 4 countries (France, Italy, UK, USA), which prove short and long term feedback hypothesis. Secondly, for the correlation flowing from import to economic growth, our findings specify that there is bidirectional temporary causality exists in Japan only, which validates the short run feedback hypothesis.

**Table 3:** Results of frequency domain granger causality test.

Study	Time period	Frequency domain GC test									
		Temporary (ῶ = 2.5)					Permanent (ῶ = 0.01)				
		Imp → Real		Real → Imp		hypothesis	Imp → Real		Real → Imp		hypothesis
China	1978–2019	8.1405	(0.0171)∗∗	2.9288	(0.2312)	ILGH	22.8867	(0.000)∗	2.6126	(0.2708)	ILGH
India	1978–2019	8.6537	(0.0132)∗∗	3.7107	(0.1564)	ILGH	8.6537	(0.0132)∗∗	3.5591	(0.1687)	ILGH
Canada	1978–2019	7.5636	(0.0228)∗∗∗	1.0835	(0.5817)	ILGH	5.8038	(0.0549)∗∗∗	1.5025	(0.4718)	ILGH
France	1978–2019	11.7964	(0.0027)∗	10.2830	(0.0058)∗	FBH	12.8167	(0.0016)∗	16.3127	(0.0003)∗	FBH
Germany	1978–2019	6.7293	(0.0346)∗∗	3.8788	(0.1438)	ILGH	17.9492	(0.0001)∗	2.3384	(0.3106)	ILGH
Japan	1978–2019	5.5818	(0.0614)∗∗∗	5.3057	(0.0705)∗∗∗	FBH	5.2724	(0.0716)∗∗∗	1.2033	(0.5479)	ILGH
Italy	1978–2019	6.5014	(0.0387)∗∗∗	13.4513	(0.0012)∗	FBH	10.7623	(0.0046)∗	7.3814	(0.0250)∗∗	FBH
UK	1978–2019	5.9146	(0.0520)∗∗∗	5.4400	(0.0659)∗∗∗	FBH	7.0211	(0.0299)∗∗	17.6749	(0.0001)∗	FBH
USA	1978–2019	4.6536	(0.0976)∗∗∗	9.6856	(0.0079)∗	FBH	14.6959	(0.0006)∗	11.6631	(0.0029)∗	FBH

Note: - Number in parentheses is p-values. ∗, ∗∗, ∗∗∗, denotes significant at 1%. 5% and 10% level respectively. Imp denotes (imports of goods and services (current US$), Real denotes (gdp per capita (constant 2010 US$).

Moreover, the unidirectional causality from imports to economic growth persists in the long run, and these findings validated the ILG hypothesis for Japan. Finally, in four countries (China, India, Canada and Germany), permanent and temporary uni-directional causal nexus from import to economic growth is noted, which strengthen the ILG hypothesis of these countries in the short and long run.

Our research strengthens the analysis of relevant aspects and studies the correlations between imports and economic growth. In addition, it expands the earlier empirical researches, because we mainly focus on China, India and G7 countries, which enables us to clearly describe the correlation between imports and economic growth of specific economies in a specific period of time.

## 6 Conclusion and limitations

As we know, the causal nexus between imports and economic growth in different regions and economies has been studied by time domain BC Granger causality method. One disadvantage of time-domain BC Granger causality test is that it fails to consider the change of causality trend between variables over time. We used the frequency domain BC granger causal method to check the causal relationship between imports and economic growth in China, India and G7. Our research results contribute in some extent to support the hypotheses such as (1). Imports-led growth (ILG) hypothesis (2). Growth-Led Imports (GLI) hypothesis (3). Feedback (FB) hypothesis (4). Neutrality (NH) hypothesis, about the causal nexus between imports and economic growth at high level frequency and low level, conforms to the short-term and long-term BC test.

In the frequency domain (high and low levels), our findings on the ILG hypothesis in 4 countries (China, India, Canada, Germany), are constant with the Maitra (2020), who examined significant results of India's short and long-term ILG hypothesis, indicating that imports have an important impact on economic growth.

In the frequency domain (high and low level), we prove the ILG hypothesis in 4 countries (China, India, Canada, Germany), however, in the short and long term, there is no indication persistent with the GLI and neutrality hypothesis compared to the research of Chang et al. (2014). Their research findings approve the unidirectional causality between growths to import in four of the nine provinces surveyed in South Africa. Aluko and &Adeyeye (2020), also observed that there is uni-directional causality between short-term and long-term economic growth and imports in several of the 41 countries surveyed but in our research there is no indication persistent with the GLI hypothesis and there is no indication persistent with the GLI hypothesis and neutrality hypothesis. The FB hypothesis in high frequency level is validate in 4 countries (France, Italy, UK and USA) and in low frequency level is proved Japan only. We notice diverse causality robustness between imports and economic growth, only in the short and long run for Japan. From our research results, we can see that in all selected countries, there is a frequency domain (high and low) proof of the bi-directional causal relationship between imports and economic growth. The imports and economic growth in most economies seem to be long and short-term dependencies.

Accordingly, this study shows that policy-makers of these big economies need to analyse the robustness of the shift in the import and economic growth cause and effect throughout the year when planning policy actions. For further study, analysts may expand the scope of analysis by investigating the causality linkage between the variables (imports and economic growth) in China, India and G7 countries and divide imports into different factors.

Regarding the methodological perspective, variations in data achieve techniques and methods, may affect the valuation of the variables. When utilizing the periodic data, it is essential to be sure that variation from one year to the following and selection of countries or region may be brought changes in the results. However, the selection of variables as a proxy (substitute) is a problematic exercise, and is still a problem. Moreover, the techniques using in this paper could modify after particular interval, suppose that periodic data in which structure breaks can be illustrated. It is critical to frequently revisit the model by comprising fresh data that could improve and modify the results.

## Declarations

### Author contribution statement

Usman Khalid: Conceived and designed the experiments; Performed the experiments; Analyzed and interpreted the data; Contributed reagents, materials, analysis tools or data; Wrote the paper.

Usman Bashir: Conceived and designed the experiments; Wrote the paper.

### Funding statement

This research did not receive any specific grant from funding agencies in the public, commercial, or not-for-profit sectors.

### Data availability statement

Data will be made available on request.

### Declaration of interest’s statement

The authors declare no conflict of interest.

### Additional information

No additional information is available for this paper.

**Appendix 1:** Level of Integration

Country	Beak in Intercept		Beak in trend		Beak in Intercept and trend (both)	
	Imp	Real	Imp	Real	Imp	Real
China	I(0)	I (1)	I (0)	I (1)	I (0)	I (1)
India	I (1)	I (0)	I (1)	I (0)	I (1)	I (0)
Canada	I (0)	I (1)	I (0)	I (1)	I (0)	I (1)
France	I (0)	I (1)	I (0)	I (1)	I (0)	I (1)
Germany	I (0)	I (1)	I (0)	I (1)	I (0)	I (1)
Japan	I (0)	I (0)	I (0)	I (0)	I (0)	I (0)
Italy	I (0)	I (1)	I (0)	I (1)	I (0)	I (1)
UK	I (0)	I (1)	I (0)	I (1)	I (0)	I (1)
USA	I (0)	I (1)	I (0)	I (1)	I (0)	I (1)
